# A novel 3D-printed noninvasive immobilizer for head stability during surgery of the orbit and skull base

**DOI:** 10.1186/s41205-026-00322-0

**Published:** 2026-04-01

**Authors:** Joseph P. Miller, Teresa H. Chen, Jeremiah P. Tao

**Affiliations:** 1 Present Address: Division of Oculofacial Plastic and Orbital Surgery, Gavin Herbert Eye Institute, University of California, Irvine, 850 Health Sciences Rd, Irvine, CA 92617 USA; 2 Department of Ophthalmology, George Washington University, Washington, D.C USA

**Keywords:** Headrest, Ophthalmology, Oculoplastic surgery, Otolaryngology, Head stability, Acceleration, 3D printing, Engineering

## Abstract

**Background:**

This study investigates a 3D printed, noninvasive headrest that was designed for use in orbital and skull base surgeries and compares the stability of this innovative headrest to conventional head immobilization devices using accelerometer-based measurements.

**Methods:**

A 3D model of a headrest was developed using two plastic materials: polyethylene terephthalate glycol (PETG) and thermoplastic polyurethane (TPU). Stability, in g-force, was measured for these headrests, Gel Head Donut Adult Blue Diamond^®^, and a no headrest condition. A non-embalmed cadaver’s head was placed in each headrest condition and subjected to controlled oscillations using Bellco Glass’ Orbital Shaker. Acceleration data were recorded over a 3-second interval.

**Results:**

The average acceleration, measured in g-force (g), over 3 s for each headrest configuration was: (1) no headrest: 0.068116 g ± 0.058498, (2) Gel Donut head immobilizer: 0.064223 g ± 0.027463, (3) PETG headrest: 0.053331 g ± 0.037782, and (4) TPU headrest: 0.056254 g ± 0.032200. The PETG headrest showed a 21.71% improvement over no headrest and a 16.96% improvement over the gel donut headrest in maintaining head stability. And the TPU headrest showed a 17.41% improvement over no headrest and a 12.41% improvement over the gel donut headrest in maintaining head stability.

**Conclusions:**

The PETG and TPU headrest provided greater stability compared to the commonly used Gel Head Donut headrest and in the absence of a headrest. This study suggests that the design of this headrest offers a potential noninvasive head stability device, regardless of material composition, that may improve the safety and efficacy of orbital and skull base surgery.

## Background

Surgery around the orbit and skull base is complex and demanding, requiring delicate manipulation and precision throughout the procedure. During surgery, keeping patients stable and reducing surgeon tremors remain areas of concern. It is known that intraoperative head drift and sudden unexpected head movements during surgery, such as in cases of snoring due to anesthesia, significantly increase risks of complications and intraocular injury [1–2]. The issue of unexpected movement is pertinent and prevalent, such that the impact of different intravenous anesthetic medications on patient motion have been investigated, in order to minimize intraoperative movements [3]. However, in the case of head immobilizers, the literature in the field of oculofacial plastic surgery is lacking.

Ophthalmic procedures require the patient to remain predictably stable and immobile, and thus various types of head immobilizers are employed. These include foam or gel donut head immobilizers, which are lightweight and conform to the occiput, providing comfort and some stability. However, under general anesthesia, most standard headrests on the market inadequately stabilize the head for higher risk orbital or skull base surgery. Some devices feature adjustable straps or foam blocks that secure the patient’s head but these interfere with surgical access and may offer limited stability [4]. Neurosurgical head immobilizers, particularly the Mayfield skull clamp, are essential tools in cranial neurosurgical and skull base procedures to ensure the patient’s head remains stable and precisely positioned throughout the surgery. The Mayfield clamp, a commonly used three-pin device, secures the head by attaching to the skull at strategic points, which helps maintain a fixed position and orientation that is crucial for successful outcomes in delicate cranial and cervical surgeries [5]. The design and application of these devices consider several factors to minimize complications and enhance surgical precision. For example, the pins used in these clamps have specific angles and clamping forces to suit different cranial structures and patient ages [5]. This careful consideration helps prevent bone damage while ensuring the head is immobilized effectively during the procedure [5].​ Modifications, such as rubber plugs or discs on the pins, have been designed to prevent excess skull damage and depression fractures [6–9]. Armstrong et al. suggested taping patient’s heads in a donut shaped headrest to reduce unintended head movement intraoperatively [10]. Other fixation devices akin to the Mayfield clamp include 3-, 4-, and 6-point fixation with a comprehensive list of pros and cons outlined in LoPresti’s et al. neurosurgery paper focusing on head fixation techniques [11]. In fact, other subspeciality surgical fields, like otolaryngology, have used modified head and neck fixation tools in order to enable surgeons to maximize surgical access while minimizing surgical field disturbances [12]. However, in orbit and many skull base surgeries, these cranial fixated immobilization devices, while potentially providing excellent stability, would be excessive and the scalp and cranial morbidity would likely outweigh the benefits [5, 13]. Moreover, set-up of these rigid head fixation devices add time that is often impractical for short duration, outpatient surgery.

There is a need for continued research and standardization in the use of head immobilizers in to ensure patient safety and minimize risks without inducing excessive restraint [14].​ Here, we introduce and compare our novel 3D printed surgical headrest against conventional and currently manufactured designs using accelerometer-based measurements as a pilot for future research and development. We aim to answer if a noninvasive 3D printable headrest device increases head stability which may fill a practical void for surgery of the orbit and skull base.

## Methods

An accelerometer device was developed to track the acceleration of the headrest during simulated movements. Four headrest configurations were tested: no headrest, the Gel Head Donut Adult Blue Diamond^®^, and our new headrest design made from two different materials; polyethylene terephthalate glycol (PETG), a hard plastic, and thermoplastic polyurethan (TPU), a soft plastic.

**Fig. 1:**
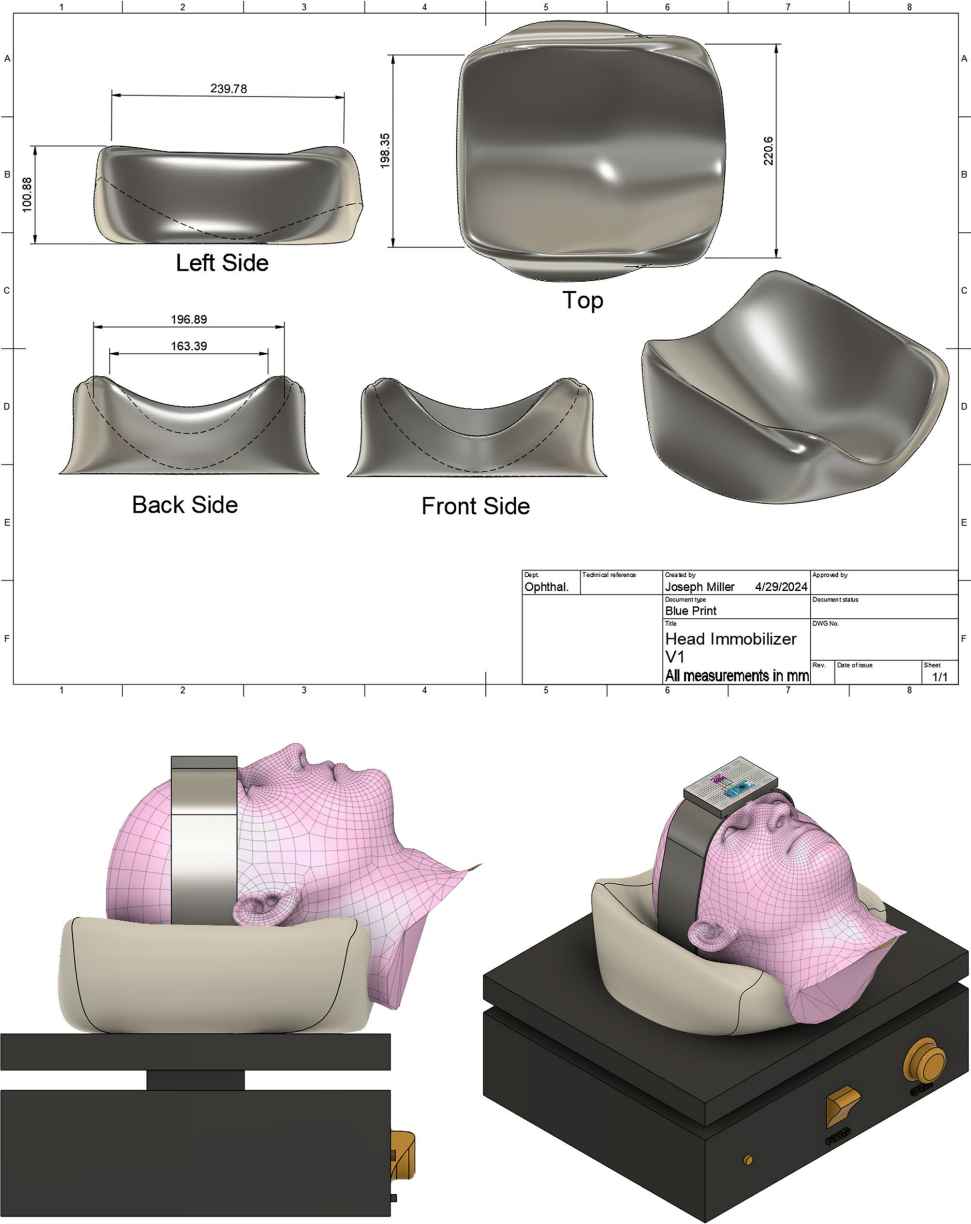
Blueprint of the headrest and Computer-Aided Design (CAD) mockup of the experimental design. This includes a mockup of where the cadaver head would rest in the 3D printed headrest on top of the Bellco Glass’ Orbital Shaker with the accelerometer fixed to the forehead with Velcro straps

The headrest designed in Autodesk Fusion (v.2.0.18961) has a top-down width of 239.78 mm, a depth of 183.95 mm from front to back, and a height of 220.6 mm (Fig. 1). The top aspect of the headrest has a smoothly contoured, rectangular shape with rounded edges, ensuring an ergonomic interaction with the head. The left side view displays a convexity peaking at 100.83 mm, which mirrors the natural curvature of the occipital region, promoting an anatomically aligned support structure. Observing the back side, a concave profile is evident, with a width of 196.9 mm and a concave profile width of 163.39 mm in the front, accommodating the posterior aspect of the head and neck respectively. This concavity transitions to the front side, which showcases a symmetrical profile designed to cradle the user’s head without imposing restrictive pressure points.

This headrest was exported from Fusion as a Standard Tessellation Language (STL) file to PrusaSlicer (v.2.4.1) for printing. We used the Prusa MK3S 3D printer for the physical creation of this headrest. Both PETG and TPU headrests had a 5% gyroid infill which optimized filament usage, structural stability, weight, and plasticity for the TPU headrest specifically. The PETG headrest took approximately 26 h to print at our optimized settings and used 189.9 g of PETG which costs approximately a total of $2.85. The TPU headrest took approximately 35 h to print at our optimized settings and used 177.9 g of TPU which costs approximately a total of $3.60. Furthermore, the individual prints for the headrest in different materials were optimized for each material, which includes printer head temperature, bed temperature, print speed, etc. When we printed with PETG, the nozzle temperature was 240 °C with a heated bed at 80 °C. To reduce stringing and improve adhesion in each layer, we printed at a slower speed of 40 mm/s. The rest of the print settings we used were the default settings in PrusaSlicer. When we printed with TPU, the nozzle temperature was also 240 °C with a heated bed at 40 °C. To reduce stringing and improve adhesion in each layer, we printed at a slower speed of 20 mm/s. The rest of the print settings we used were the default settings in PrusaSlicer. For both prints, the print bed surface was textured polyetherimide (PEI) and we did not use any chemicals or adhesion reduction materials on the surface of the bed. However, it would be reasonable to do so to prevent the resulting print from being overly adhered to the bed plate and requiring excessive force to remove. Also, we printed with newly opened spools of filament to optimize the print as most filaments can and will absorb water from the atmosphere which negatively affect the resulting prints. Our filament was stored in and printed from dry boxes to reduce this water absorption from occurring.

Two separate whole body, non-embalmed cadaver heads were used in this study (biological replicates). For each cadaver, acceleration data were recorded twice per headrest condition, resulting in two technical replicates per condition per cadaver. The non-embalmed cadavers’ heads were placed in each headrest condition and then subjected to controlled oscillations using Bellco Glass’ Orbital Shaker. (SKU: 7744 − 02020) set to a speed of 3 out of 10 on the dial before powering on. Of note, Bellco does not publicly publish official data or spec sheets mapping the dial numbers to exact revolutions-per-minute (RPM) values. Rather it is just a relative position within the range of 10–350 RPM range. The orbital shaker was positioned beneath the cadaver head, with and without the head resting in the headrests depending on the condition tested. The cadaver was placed supine such that the whole cadaver body and head rested parallel to the ground. The shaker generated circular motion in the transverse plane in space (or the coronal plane relative to the cadaver head anatomically). This accelerometer was affixed directly to the midline forehead using Velcro straps (demonstrated in Fig. 1). The acceleration data were recorded twice per headrest condition per head, each over a 3-second interval for each headrest configuration recording 618 g-force recordings per headrest condition. The accelerometer’s individual parts included a small breadboard, Arduino nano, and 3-axis accelerometer/gyroscope sensor module (GY-521 MPU-6050) that ran a custom code which enabled the accelerometer to record approximately 206 data points per second. We tested the headrest types in the following order: (1) No headrest; (2) Donut headrest; (3) PETG headrest; (4) TPU headrest. Also, for this experiment, the non-embalmed cadaver is in secondary flaccidity or the flexible stage after rigor mortis and conducted in a short continuous session, minimizing progressive tissue changes.

All statistics were calculated via python script. This includes collecting and visualizing acceleration data via custom scripts, and the statistical analysis of each headrest acceleration change over time. Averages, standard deviation, analysis of variance (ANOVA) with a Tukey Honesty Significant Difference (HSD) post-hoc analysis, ANOVA F-statistic (or F-stat), and pairwise t-testing were performed on this data. Parametric tests were selected due to the large number of high-frequency acceleration measurements per condition, allowing approximation of normality via the central limit theorem. However, significance is limited in this pilot study due to the small number of independent biological samples (*n* = 2 cadaver heads), and results should be interpreted with caution. Python script of the statistical analysis can be found via the following link: [https://github.com/shabopbop/3D-Printed-Headrest-Python-Script-for-Statistical-Analysis.git].

## Results

**Fig. 2:**
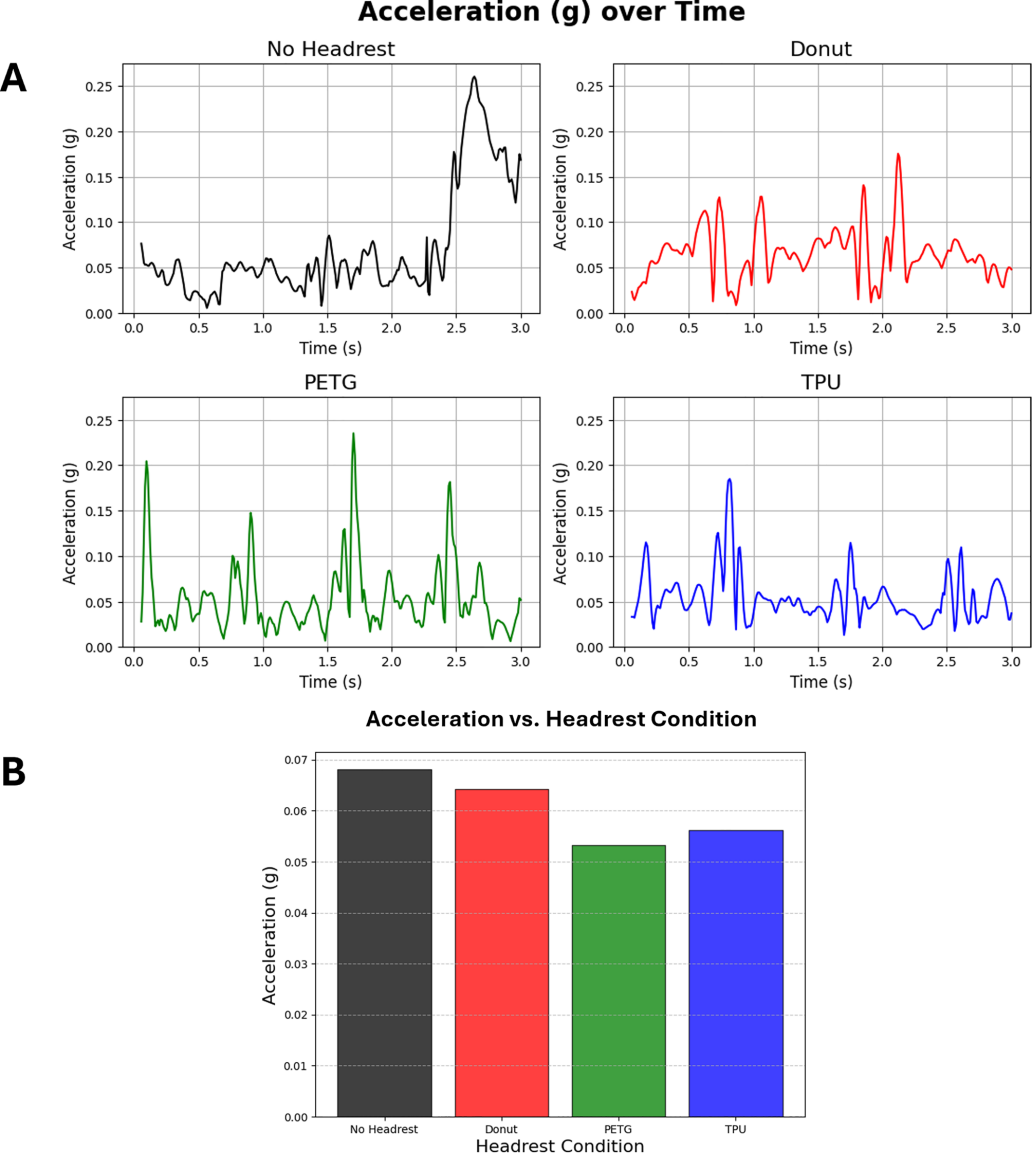
Average acceleration tracked and averaged. **A** shows the actual tracked change in acceleration throughout the time course of one experiment. **B** shows the overall average acceleration that was experienced in all experiments performed in this study. No significance can be determined due to low sample size

The average absolute value of acceleration, in g-force (g), over 3 s for each headrest configuration was as follows: (1) no headrest: 0.068116 g ± 0.058498, (2) Gel Donut head immobilizer: 0.064223 g ± 0.027463, (3) PETG headrest: 0.053331 g ± 0.037782, and (4) TPU headrest: 0.056254 g ± 0.032200 (Table 1; Fig. 2b). An ANOVA comparison between headrest conditions showed an F-stat of 19.243125 and an ANOVA p-value of 2.396451e-12 (Table 2). Tukey HSD post-hoc analysis showed adjusted p-values (or p-adj values) between no headrest and Gel Donut, no headrest and PETG headrest, and no headrest and TPU headrest equaling 0.2872, < 0.0001, and < 0.0001 respectively (Table 2). P-adj values between the Gel Donut and PETG headrest, and Gel Donut and TPU headrest equaling < 0.0001 and 0.0008 respectively (Table 2). And the p-adj value between PETG headrest and the TPU headrest was 0.5277 (Table 2). It is important to note that our Tukey HSD post-hoc library in our script automatically limits its calculations of the p-adj value where if the number is astronomically small, it will round the number down to 0 to preserve efficiency. This means that the p-adj values between the no headrest vs. the PETG and TPU headrests were not actually 0, but rather infinitesimally small and rounded down to 0.

**Table 1:** Data summary of head acceleration

Condition	Mean (g)	STD (g)	Min (g)	Q1 (g)	Q2 (g)	Q3 (g)	Max (g)
No Headrest	0.068116	0.058498	0.005554	0.034668	0.048157	0.067078	0.260376
Donut	0.064223	0.027463	0.008606	0.04805	0.062378	0.076111	0.175415
PETG	0.053331	0.037782	0.006409	0.029297	0.043457	0.060791	0.235168
TPU	0.056254	0.032200	0.010315	0.037003	0.047852	0.064941	0.228088

This table summarizes the raw acceleration values, in g-force (g), recorded from two separate cadaver heads across each headrest condition. For each cadaver, acceleration was measured twice per headrest condition when placed on the orbital shaker. Each trial consisted of high-frequency recordings over a 3-second interval. Reported values reflect the distribution of all recorded data points. Q1, Q2 (median), and Q3 represent the 25th, 50th, and 75th percentiles, respectively

**Table 2:** ANOVA results and Tukey Post-hoc analysis of our acceleration data

		ANOVA F-stat		ANOVA *p*-value	
		19.24312463		2.39645E-12	
Condition 1	Condition 2	*p-adj*	*Mean difference*	*lower*	*upper*
No Headrest	Donut	0.2872	-0.0039	-0.0018	0.0095
No Headrest	PETG	< 0.0001*^α^	-0.0148	-0.0206	-0.009
No Headrest	TPU	< 0.0001*^α^	-0.0119	-0.0173	-0.0064
Donut	PETG	< 0.0001*^α^	-0.0109	-0.0166	-0.0052
Donut	TPU	0.0008*	-0.008	-0.0133	-0.0026
PETG	TPU	0.5277	0.0029	-0.0026	0.0085

Analysis includes the analysis of variance (ANOVA) results, and the following Tukey post-hoc analysis results. This includes a p-adj value, or an adjusted p-value, that corrects the original p-value to account for the multiple hypothesis testing to reduce the risk of false positives. The difference of the means and lower and upper limits of the difference is also included

Note: Within each dependent measure, p-adj values with an asterisk (*) indicate significance levels of *p* < 0.001. P-adj values with an alpha symbol (α) indicate a resulting value due to a limitation in our Tukey post-hoc analysis script. Tukey HSD post-hoc library in our python script automatically limits its calculations of the p-adj value where if the number is astronomically small, it will assign the number down to < 0.0001 to preserve computational efficiency

A follow up pairwise T-test showed p-values between no headrest and Gel Donut, no headrest and PETG headrest, and no headrest and TPU headrest equaling 0.122711, 9.37e-8, and 4.12e-6 respectively (Table 3). P-values between the Gel Donut and PETG headrest, and Gel Donut and TPU headrest equaling 4.77e-9 and 2.97e-7 respectively (Table 3). And the p-value between PETG headrest and the TPU headrest was 0.124461 (Table 3).

**Table 3:** Pairwise t-test results with included bonferroni adjustment

Condition 1	Condition 2	t-stat	*p*-value	*p*-value-Bonferroni
No Headrest	Donut	1.544927	0.122711	0.736266
No Headrest	PETG	5.373919	9.37E-08*	5.62E-07*
No Headrest	TPU	4.631739	4.12E-06*	2.47E-05*
Donut	PETG	5.901658	4.77E-09*	2.86E-08*
Donut	TPU	5.148838	2.97E-07*	1.78E-06*
PETG	TPU	-1.53738	0.124461	0.746767

This analysis shows the p-value of t-tests between the indicated headrest conditions. A Bonferroni correction was also calculated to reduce the risk of false positives when conducting multiple statistical tests

Note: Within each dependent measure, p-adj values with an asterisk (*) indicate significance levels of *p* < 0.001

The PETG headrest showed a 21.71% improvement over no headrest and a 16.96% improvement over the gel donut headrest in maintaining head stability. And the TPU headrest showed a 17.41% improvement over no headrest and a 12.41% improvement over the gel donut headrest in maintaining head stability.

## Discussion

The results indicate that both PETG and TPU headrest provided greater stability compared to the commonly used gel donut headrest and compared to the absence of a headrest. The PETG headrest demonstrated the lowest average acceleration values, indicating superior stability in mitigating simulated head movement. However, the TPU headrest showed greater, but non-significant, acceleration values that were only slightly higher than the PETG, suggesting comparable performance in stability. These findings highlight the need for further experimentation, particularly considering patient comfort when selecting the material composition for the headrest. If both headrests provide similar stability outcomes, prioritizing comfort could play a key role in determining the optimal design.

Of note, our data shows a lot of variation in the No Headrest condition when running this test, as indicated by the standard deviation (Table 1). Additionally, there was a large increase in acceleration in the no headrest condition towards the end of the 3 seconds in both experiments (Fig. 2a), likely due to the cadaver head falling over to the side due to the lack of support keeping the head in a face up position that a patient would have during an actual procedure with other equipment. This lack of support is assumed to be the cause of such large variation and shows how unstable a non-fixed head is when there is a lack of muscular or structural support. Such structural support during a procedure of course would include arm boards, positioning straps, gel pads, and foam wedges for the body.

In addition to the mechanical stabilization provided by devices like the Mayfield clamp, patient positioning plays a critical role. Proper positioning can prevent pressure-related injuries, facilitate surgical access, and reduce surgeon fatigue. This involves not only the immobilization of the head but also the strategic positioning of the entire body to provide the best possible access to the surgical site while keeping the patient’s physiological needs in mind [15, 16].​ Further studies that would provide more insight to maximize stabilization would be to examine other various types of external immobilizers that restrict head and cervical spine movement. The addition of a cervical collar, cervico-thoracic, or cranio-thoracic devices, for example, may help with reducing the range of motion for patients during and post-surgery [17]. However, patient preparation in wearing these devices would increase surgical preparation time, complicate equipment usage, and provide potentially minimal benefit. Thus, diving into this design may help elucidate a new ophthalmological protocol for surgery.

Patients undergoing procedures may also have long hair, and managing the patient’s hair introduces another practical issue with this headrest design as it does not accommodate hair bulk (such as ponytails or buns). Future iterations of the headrest may include variable depth bowls, adjustable modular inserts based on hair profile, a central posterior channel to accommodate hair bulk, or a removable occipital insert. It may be worth looking into having patients wear a Lycra or silicone cap (similar to a swim cap) to conform hair closer to the natural shape of the head when using this device in order to form a better fit and better distribution of pressure points while resting in the headrest.

We set out to design this headrest with a broad concave contour to distribute force over the occiput rather than focal contact points. However, pressure mapping was not performed in this pilot study. Future studies should include pressure mapping and pressure analysis between headrest conditions. Furthermore, future studies should also include 3D scanning individual head contours to see if creating personalized headrests distributes force over the contours of the headrest more evenly than a manually designed headrest.

To continue on that topic, it may be of interest to further this research by optimizing customization. A major advantage for 3D printing is that you can quickly create custom components. In this project, one headrest iteration was designed with rough measurements based off one of the author’s head shapes. However, we did notice that when a different author lay in the headrest, it was too small for them to comfortably rest their head in. Due to this, we opted to design a more generic headrest based on the combination of head shapes we had as a group; however, this opened the door to discussing further design improvements and implementation in the next iteration of this project. In this context, there are many 3D scanners on the market that we could use to scan the back of an individual’s head to get the perfect concavity for their specific head shape. Essentially, this would allow for customized headrests that we could print in clinic in about one day for that specific patient which, in theory, would maximize head stability and comfort. And we aim to pursue this idea in the future.

One important critique to address is the oscillation model of movement we analyzed. The oscillation was intended to provide standardized, reproducible perturbation for relative comparison across headrest devices, and not intended to replicate specific intraoperative movement amplitudes. We acknowledge that sudden patient movements (like coughing, snoring, anesthesia drift, etc.) may produce larger non-periodic forces and future work should evaluate sudden impulse forces, surgeon-applied manipulation forces, and jerk (rate of change of acceleration).

Another avenue that would be interesting to explore is to determine if certain subspecialties of ophthalmology benefit more from the use of a head stabilizing device than others. For example, an oculofacial plastic surgeon whose surgery focuses on the eyelids and the orbit may find having a head stabilizer more useful than other ophthalmic subspecialties that perform surgery only on the eyeball itself, as the oculofacial plastic surgeries require more manipulation on the face and head. It also seems to be appropriate for shorter, lower-risk anterior skull base cases where rigid fixation is not indicated such as some endonasal endoscopic procedure. It has the potential to be useful for any procedures that would otherwise use a donut headrest. The only surgeries that this specific design would not be applicable would be prone or side lying positions. To emphasize, this headrest is not intended to replace pin fixation for complex cranial neurosurgical procedures requiring absolute immobilization.

In conclusion, this novel geometric configuration that is designed to fit to the occiput of the head achieves high head stability in these experiments. PETG and TPU material performed optimally, and almost equally, in stabilizing the cranium relative to both no headrest and a commonly used Gel Donut head stabilizer. While the subject sample size can be increased for larger statistical power, the design and material attributes identified in this pilot study offers a potential noninvasive head stability device that may improve the safety and efficacy of orbital and skull base surgery. Of note, it seems that the density of material is less important for absorbing excess force and reducing acceleration change than the contour design itself of the headrest. Determining patient comfort, surgeon satisfaction, jerk measurement, and adding a larger sample size are indicated in future experiments with this specific device. Optimizing the curvature of the bowl of the device to the posterior head contours of individual patients may also lead to further stability optimization; this includes changing the size of the device to correlate to patient cranium sizes and bowl contours to contour to the specific shape of the head for each patient. Finally, compared to the commonly used adult sized Gel Head Donut medical headrest, our designed headrest not only stabilized the cranium more, but also costs a fraction of the price. Our production costs of the PETG and TPU headrests cost $2.85 and $3.60 respectively. The retail price for the Gel Head Donut Adult Blue Diamond ^®^ is approximately $73–102 (price range gathered from Omega Surgical Supply, David Scott, and USA Medical Surgical websites). While this comparison assumes access to a 3D printer, the significant cost savings in creating a head-stabilizing product, combined with the added benefits of improved stabilization and the ability to customize the contours of the 3D-printed headrest to match an individual’s occipital shape, make it a compelling product for further exploration and study.

## Data Availability

The dataset(s) supporting the conclusions of this article is(are) available in the Science Data Bank repository, [https://www.scidb.cn/en/s/6vA32m]. Also, the Python script for data analysis can be found on Github with the following link [https://github.com/shabopbop/3D-Printed-Headrest-Python-Script-for-Statistical-Analysis.git].

## Abbreviations

STL: Standard Tessellation Language
PETG: Polyethylene terephthalate glycol
TPU: Thermoplastic polyurethan
PEI: Polyetherimide
HSD: Honesty significant difference
ANOVA: Analysis of variance
F-stat: F-statistic
RPM: Revolutions-per-minute
